# Potentially Inappropriate Medication Use in Patients with Dementia

**DOI:** 10.3390/ijerph191811426

**Published:** 2022-09-10

**Authors:** Kyungwon Yoon, Jung-Tae Kim, Won-Gun Kwack, Donghyun Kim, Kyung-Tae Lee, Seungwon Yang, Sangmin Lee, Yeo-Jin Choi, Eun-Kyoung Chung

**Affiliations:** 1 Department of Pharmacy, College of Pharmacy, Kyung Hee University, Seoul 02447, Korea; 2 Department of Pharmacy, Kyung Hee University Hospital, Seoul 02447, Korea; 3 Department of Pharmacy, Kyung Hee University Hospital at Gangdong, Seoul 05278, Korea; 4 Division of Pulmonary, Allergy and Critical Care Medicine, Kyung Hee University Hospital, Seoul 02447, Korea; 5 Department of Biomedical and Pharmaceutical Sciences, Graduate School, Kyung Hee University, Seoul 02447, Korea; 6 Department of Pharmaceutical Biochemistry, College of Pharmacy, Kyung Hee University, Seoul 02447, Korea; 7 Department of Regulatory Science, College of Pharmacy, Graduate School, Kyung Hee University, Seoul 02447, Korea

**Keywords:** potentially inappropriate medication, geriatrics, dementia, Beers criteria, polypharmacy

## Abstract

The objective of this study was to characterize the epidemiology of using potentially inappropriate medications associated with dementia exacerbation (DPIMs) in elderly outpatients with dementia. Electronic medical records were retrospectively reviewed for geriatric patients with dementia who were prescribed at least one medication in 2016 at a tertiary, university-affiliated hospital. The 2015 Beers criteria were used to define DPIMs. Logistic regression was performed to identify factors associated with prescribing DPIMs in patients with dementia. Among 2100 patients included in our study, 987 (47.0%) patients were prescribed at least one DPIM. Benzodiazepines were the most frequently prescribed DPIM followed by anticholinergics, histamine H2-receptor blockers, and zolpidem. The risk of prescribing DPIMs was significantly increased in female patients (odds ratio (OR) 1.355) with polypharmacy (OR 5.146) and multiple comorbidities (OR 1.129) (*p* < 0.05 for all). Coexistence of Parkinson’s disease (OR 1.799), mood disorder (OR 1.373), or schizophrenia (OR 4.116) in patients with dementia further increased the likelihood of receiving DPIMs. In conclusion, DPIMs were commonly used in elderly patients with dementia in Korea with benzodiazepines most frequently prescribed followed by anticholinergics. Female patients using polypharmacy with multiple comorbidities should be closely monitored to minimize unnecessary DPIM use and, ultimately, DPIM-related harms.

## 1. Introduction

As the global population ages, the proportion of individuals aged 65 years or older, defined as the geriatric population, has been exponentially and rapidly growing [1]. Aging is associated with substantial physiologic alterations, which makes elderly patients a vulnerable, special population for appropriate pharmacotherapy [2,3,4]. Advanced age is a risk factor for many chronic disorders including cardiovascular disease, diabetes mellitus, and neurological diseases; thus, the prevalence of chronic diseases is higher in geriatric patients, resulting in a subsequent increase in healthcare utilization and medication use [5,6]. One of the crucial concerns in geriatric pharmacotherapy is polypharmacy (i.e., ≥5 concomitant medications administered daily) [7,8]. The prevalence of polypharmacy among patients at the age of 65 years or older is at least 40% to 50%, potentially increasing the risk of drug–drug interactions and consequent adverse outcomes such as repeated hospitalizations [7,9,10]. In addition to polypharmacy as well as multiple comorbidities in the elderly, physiologic alterations associated with aging itself contribute to pharmacokinetic and pharmacodynamic changes, leading to an increased risk for adverse drug events (ADEs) [7,11]. Therefore, appropriate medication use by thoroughly evaluating the benefits and risks of each drug is critical in elderly patients to improve treatment outcomes as well as to prevent ADEs.

In an effort for rational drug therapy, medications whose potential risk outweighs the potential benefits in the geriatric population were defined as potentially inappropriate medications (PIMs) [12,13]. One of the most commonly used criteria to identify PIMs for older people in clinical practice is Beers criteria which has been periodically updated and expanded by the American Geriatrics Society since its original development in 1991 [14,15]. Risks associated with using a PIM in the elderly include cognitive impairment, delirium, falls, and fractures, potentially leading to clinically significant harmful events such as hospitalizations, emergency department (ED) visits, and even death [12,16]. According to a previous retrospective study, use of at least one PIM substantially increases the likelihood of hospitalization and ED visits by 2.25- and 1.59-fold, respectively, in the elderly population [17]. However, PIMs have been frequently used in older individuals as suggested by previous studies reporting at least 40% to 50% of geriatric patients prescribed one or more PIM in clinical practice [16,18,19].

Dementia is one of the most common geriatric diseases with its prevalence rapidly increasing over the last couple of decades [20,21]. Although the etiology of dementia may differ among the patients, the majority of patients with dementia have multiple comorbidities such as depression and Parkinson’s disease, usually requiring treatment with multiple medications including PIMs [22,23,24,25]. As a result, patients with dementia might be at an increased risk of PIM-induced cognitive impairment such as impairments in language, working memory, and processing speed and other clinically significant events including increased risks of falls, physical dysfunction, and delirium [26,27], consequently contributing to increased mortality as well as accelerated disease progression as indicated by reduced scores of Mini-Mental State Examination (MMSE) [15,22,28]. Due to the substantial risk of certain PIMs in patients with dementia, the specific Beers criteria for dementia were developed based on the risk of drug-disease interaction that may exacerbate dementia as the pharmacokinetic and pharmacodynamic characteristics change with dementia progression [29,30]. According to a previous study evaluating the use of PIMs associated with dementia exacerbation (DPIMs) in eight European countries, 60% of patients with dementia were prescribed at least one PIM. However, little is known regarding DPIM use in patients with dementia in Korea. Considering the variability in demographic, clinical, and sociocultural aspects associated with drug therapy and disease states across different countries, specific data regarding the epidemiology of DPIMs in each country may be helpful for establishing optimal strategies to prevent excessive use of DPIMs [31]. Therefore, the aims of this study were to investigate the prevalence of using at least one DPIM for ambulatory patients with dementia of any cause in a tertiary, university-affiliated hospital in Korea and to identify factors associated with prescribing at least one DPIM in patients with dementia.

## 2. Materials and Methods

### 2.1. Study Design

This study was conducted by retrospectively reviewing electronic medical records (EMRs) of patients with dementia who had at least one documented outpatient prescription for systemic use from 1 January 2016 to 31 December 2016 at Kyung Hee University Hospital (Seoul, Korea), a tertiary academic medical center with 850 beds. Patients at the age of 65 years or older were included if they were diagnosed with dementia of any cause including Alzheimer’s disease based on the International Classification of Diseases (ICD-10). According to the ICD-10 codes, dementia was defined as the diagnostic code of F00 (dementia in Alzheimer disease with late onset), F01 (vascular dementia), F02 (dementia in other disease classified elsewhere including Creutzfeldt-Jakob disease, Huntington’s disease, Parkinson’s disease, and autoimmune or infection-related dementia), and F03 (unspecified dementia) including all subcategories of each disease code [32]. This study was approved by the Institutional Review Board of Kyung Hee University Hospital (approval No.2017-07-068-004), and informed consents were exempted by the board.

### 2.2. Data Collection, Definition, and Analyses

The following demographic and clinical information was retrieved for each patient from EMRs: age; gender; comorbid conditions including Parkinson’s disease (G20-22), mood (affective) disorders (F30-39) such as bipolar disorder and depression, schizophrenia (F20-29), and gastrointestinal disorders (K20-31); prescribed medications; and duration of medication administration. In this study, DPIM was defined by the 2015 Beers criteria as PIMs in the elderly patients due to drug-disease interactions that may exacerbate dementia [15]. Drug classes included in this study as DPIMs were anticholinergics, benzodiazepines, histamine H2-receptor antagonists (H2RAs), and nonbenzodiazepine/benzodiazepine receptor agonist hypnotics (i.e., zolpidem). Based on the formulary of our institution, medications defined as DPIMs are listed in Table 1. Reasons for prescribing DPIMs were identified as the indicated comorbid condition using the ICD-10 codes based on the approved label for each DPIM. Patients prescribed 2 or more DPIMs were classified as multiple DPIM users considering substantially elevated risk of adverse health outcomes in patients who administered at least 2 PIMs [33]. The relative quantity of DPIM use in each patient was analyzed to assess the extent of DPIM usage in dementia management during the study period as considerable amount of DPIM usage makes patients susceptible to adverse outcomes [34] The relative quantity (%) of DPIM use over the study period was calculated as follows:

Relative quantity of DPIM use (%)=[(Number of DPIMsDose)×(Number of dosesDay)×(Total days of treatment)][(Number of all medicationsDose)×(Number of dosesDay)×(Total days of treatment)]×100

### 2.3. Statistical Analysis

All statistical analyses were performed using SPSS Statistic 23.0 (version 23.0; IBM SPSS Statistics for Windows, Armonk, NY, USA). Descriptive statistics were used to estimate the prevalence of DPIM use. Comparison between patients with at least one DPIM prescription and those who did not use any DPIM was performed utilizing Mann-Whitney U test for non-normally distributed continuous data, unpaired 2-tailed *t* test for normally distributed continuous data, and the χ^2^ or Fisher’s exact test for categorical data. Normality for continuous variables was assessed by the Kolmogorov-Smirnov test.

In order to identify factors significantly associated with DPIM use, the univariate analysis was first performed for the following factors: age, sex, number of concurrent medications, number of comorbid diseases, and presence of each concomitant disease. Multiple logistic regression was then conducted using a stepwise forward method by examining the factors significantly associated with prescribing PIMs in patients with dementia in the univariate analysis. Odds ratios (ORs) with the corresponding 95% confidence intervals (CIs) were estimated, and any *p*-value < 0.05 was considered statistically significant.

## 3. Results

### 3.1. Baseline Characteristics

A total of 2100 patients with dementia satisfied the eligibility criteria of this study. Baseline characteristics of patients included in this study are described in Table 2. Overall, the majority of patients included in this study were females (*n* = 1326; 63.1%). The median age of included patients was 77 years (interquartile range (IQR) of 73–81), and prevalent polypharmacy was observed with the median number of concurrent medications of 6 (IQR 3–9). All patients had at least one additional comorbid disease (IQR for the number of comorbid diseases, 3 to 7), most frequently mood disorder (*n* = 740; 35.2%) followed by Parkinson’s disease (*n* = 518; 24.7%).

### 3.2. Prevalence of DPIM Use

Table 2 and Table 3 summarize characteristics of DPIM users and the overall prevalence of DPIM use in our study patients, respectively. Among the 2100 patients included in our study, 987 (47.0%) patients were prescribed at least one DPIM. Compared to DPIM non-users, DPIM users were more likely to be female patients, have more comorbidities, and use more concurrent medications (Table 2). Multiple DPIMs were prescribed to 432 (20.6%) patients (Table 3). The median relative quantity of DPIM use over all medications administered to dementia patients during the study period was 14.3% with an IQR of 8.1% to 24.2%. The most frequently prescribed DPIM was a benzodiazepine (*n* = 601; 28.6%) followed by anticholinergics (*n* = 555; 26.4%). H2RAs and zolpidem were prescribed to 146 (7.0%) and 92 (4.4%) patients, respectively.

### 3.3. Factors Associated with DPIM Use 

The univariate analysis showed significant association of DPIM use with sex; age; polypharmacy use; number of comorbid diseases; and the presence of Parkinson’s disease, mood disorder, schizophrenia, and gastrointestinal disorder (*p* < 0.05, Table 4). Based on the multivariate regression analysis, DPIMs were more likely to be prescribed in the following (Table 4): female patients (OR 1.355, 95% CI 1.103–1.664, *p* = 0.004), individuals using polypharmacy (OR 5.146, 95% CI 3.912–6.768, *p* < 0.001), patients with multiple comorbidities (OR 1.129 for each one additional comorbid disease, 95% CI 1.073–1.188, *p* < 0.001), those with Parkinson’s disease (OR 1.799, 95% CI 1.416–2.286, *p* < 0.001), those with mood disorder (OR 1.373, 95% CI 1.111–1.695, *p* < 0.001), and those with schizophrenia (OR 4.116, 95% CI 1.959–8.648, *p* < 0.001).

## 4. Discussion

We evaluated the prevalence and risk factors of prescribing DPIMs in patients with dementia over one year at a tertiary academic medical center in Korea. The prevalence of using PIMs possibly exacerbating cognitive function in patients with dementia was relatively high (47%) (Table 2 and Table 3). All patients included in our study took multiple medications (Table 2), and one in seven medications was a DPIM (Table 3). Moreover, the median relative quantity of DPIM use during the study period in our study population was 14.3% (IQR 8.1–24.3). The relative quantity of DPIM use escalated with advancing age, with the highest quantity prescribed to patients over 80 years of age; this suggests DPIMs commonly prescribed to patients with dementia. Furthermore, more than one in five patients included in our current study were prescribed multiple DPIMs (Table 3), which might further increase the risk of exacerbating dementia [35,36]. Our current study findings are consistent with previous studies suggesting a relatively high frequency of using PIMs in patients with neuropsychiatric comorbidities including dementia as well as epilepsy, depression, delirium, insomnia, and Parkinson’s disease [37,38]. This is of crucial concern in patients with dementia because multiple neuropsychiatric diseases frequently coexist in these patients, and several medications commonly used to treat these disorders are considered DPIMs based on the Beers criteria. This high prevalence of DPIM use in a relatively large quantity (Table 3) might result from insufficient knowledge of clinicians in geriatric pharmacotherapy and extremely busy clinical setting which significantly limits time commitment to appropriate medication therapy [39,40]. Considering the rapid increase in the global prevalence of dementia projected to be >15% of geriatric patients by 2050 [31], DPIM use might further accelerate dementia progression [35,36,41], contributing to substantial dementia-related burden to the society as well as to the healthcare systems.

In this study, DPIMs were more likely to be prescribed to female patients with multiple comorbid diseases using polypharmacy (Table 4). Consistent with our present study, previous studies suggested a higher likelihood of using PIMs in female geriatric patients including those with dementia [10,42,43], possibly associated with sex-related comorbidities [44,45,46]. Compared to males, female patients are more vulnerable to developing anxiety, insomnia, incontinence, depression, and other mood disorders [16,47,48]. These are common indications for using benzodiazepines, hypnotics, and anticholinergics, which are listed as DPIMs based on the Beers criteria [15]. Similar to previous studies [16,49,50,51,52,53,54], polypharmacy was a significant risk factor for prescribing PIMs in patients with dementia, possibly due to the presence of certain comorbidities associated with polypharmacy such as Parkinson’s disease, mood (affective) disorder, and schizophrenia. Thus, patients with dementia requiring multiple medications for treatment of comorbidities should be more closely monitored. Notably, female sex, polypharmacy, and multiple comorbidities are known risk factors of dementia progression [46,55,56]. This might imply the risk of dementia progression in female patients using five or more medications to treat multiple concomitant diseases might be attributed to DPIM usages [57,58]. Future studies are warranted for effective medication management including medication reconciliation and deprescribing to prevent rapid progression of dementia as well as to reduce potential harms caused by PIMs in elderly patients with dementia in the real-world setting.

Our current study suggested benzodiazepines as the most commonly prescribed DPIM class in patients with dementia, followed by anticholinergics (Table 3). Zolpidem was the most frequently used DPIM as a single agent. These medications are commonly prescribed to manage various comorbid conditions in patients with dementia including anxiety, agitation, delirium, insomnia, psychosis, and Parkinsonism [59,60,61,62]. Consistently, our present study showed a significantly increased risk of prescribing DPIMs in the presence of these comorbidities, i.e., Parkinson’s disease, mood (affective) disorder, and schizophrenia (Table 4). Benzodiazepines, anticholinergics, and zolpidem may cause cognitive impairment, falls, and fractures, potentially contributing to mortality and morbidity associated with these drugs [9,63,64,65,66]. The risk for ADEs associated with benzodiazepines, anticholinergics, and zolpidem is especially high in geriatric patients due to increased pharmacodynamic sensitivity to these medications as well as decreased pharmacokinetic elimination of these drugs with aging [67]. This is of particular concern in patients with dementia due to adverse effects of benzodiazepines, zolpidem, and anticholinergics on cognitive function, potentially resulting in accelerated dementia progression [15,68,69,70,71]. In addition, recent studies suggested benzodiazepines and zolpidem induce significant non-cognitive functional decline attributed to pneumonia and hip fractures in patients with dementia, substantially elevating the risk of morbidity and mortality [72,73,74,75]. Nonetheless, the global prevalence of using benzodiazepines, anticholinergics, and zolpidem in elderly patients is still high [16,47,59,76,77,78], accounting for >25% of all PIM usages. Systematic interventions are urgently needed in clinical practice for promoting awareness of appropriate geriatric pharmacotherapy as well as implementing inter-professional medication review and management to avoid unnecessary DPIM use in patients with dementia.

In contrast, gastrointestinal disorders were not significantly associated with DPIM use (Table 4). Among our study patients with gastrointestinal disorders (*n* = 407; 19.4%), H2RAs were prescribed to only approximately one third of them (*n* = 146; 7.0%) (Table 2 and Table 3). Gastrointestinal disorders are common indications for H2RA use; however, the availability of therapeutic alternatives to H2RAs including proton pump inhibitors (PPIs) and potassium-competitive acid blockers (i.e., prazans) might account for the relatively low rate of H2RA use among study patients as well as the lack of association between gastrointestinal disorders and DPIM use [79]. This suggests the majority of patients with gastrointestinal disorders receive alternative therapy other than H2RAs. In the 2015 Beers criteria, H2RAs were listed as DPIMs to avoid in elderly patients with dementia because of the risk of cognitive decline [15,70,80]. However, recent studies suggested the lack of strong evidence for the association between H2RAs and impaired cognitive function [70,81]; most of the H2RA adverse effects on the central nervous system such as cognitive impairment and delirium occur at supratherapeutic doses, particularly in patients with reduced kidney function receiving conventional doses not adjusted for the renal function [70]. Furthermore, some studies suggested increased risk of cognitive impairment or dementia progression with PPI use [82]; therefore, considering adverse outcomes such as increased risk of fractures and infections, chronic PPI use in the elderly may not be a favorable treatment option [83].Therefore, H2RAs were removed from the DPIM list in the 2019 updated Beers criteria due to weak evidence on H2RA-induced cognitive impairment, thereby avoiding excessively limited therapeutic options for the elderly dementia patients with gastrointestinal diseases [70]. However, the updated Beers criteria still recommends dosage reduction in patients with renal insufficiency to avoid mental status changes [70].

There are some study limitations to be addressed. First, this was a single-center, retrospective study conducted by reviewing outpatient EMRs over the period of one year in 2016. Considering the variability in medication use across different practice sites as well as different countries along with the updated Beers criteria in 2019, caution should be exercised when interpreting and generalizing our study findings. In addition, due to the retrospective nature of this study based on outpatient EMRs, our study results were primarily dependent on documented clinical data in the EMR. For the same reason, we classified Parkinson’s disease as a separate comorbidity in patients with Parkinson’s disease dementia despite strong association between Parkinson’s disease and Parkinson’s disease dementia. Additionally, benzodiazepines are commonly prescribed to patients with various psychiatric disorders including insomnia and anxiety. However, they were frequently prescribed in dementia without separate diagnostic codes for insomnia or anxiety as they are common presenting symptoms in dementia. In this study, the majority of our patients did not have those separate diagnostic codes for benzodiazepine use. Hence, other clinical indications for benzodiazepine use than dementia could not be recorded in this study. Furthermore, appropriateness of antipsychotics without strong anticholinergic activity as well as other undocumented comorbid diseases that might have contributed to DPIM were not assessed. According to the Beers criteria, antipsychotic use in patients with dementia is generally considered inappropriate due to the risk of stroke and mortality [15,70]. However, antipsychotics without strong anticholinergic activity may be considered appropriate if nonpharmacologic therapy has failed or is not feasible and the patient is substantial threat to self or others [15,70]. Despite comprehensive EMR review, only limited information was available to determine the appropriateness of using antipsychotics without strong anticholinergic activity in our present study. Lastly, outcomes on progressive cognitive impairment associated with DPIM use were not evaluated owing to the lack of sufficient information documented in the EMR. Nonetheless, our current study might contribute to the current body of literature regarding epidemiological features of PIM use in dementia using EMR-based real-world data including medications often prescribed without insurance reimbursement in Korea (e.g., benzodiazepines, zolpidem). Further research investigating clinical outcomes associated with using DPIMs including antipsychotics with a larger sample size is warranted to optimize pharmacotherapy in geriatric patients, particularly those with dementia.

## 5. Conclusions

In conclusion, the prevalence of DPIM use in elderly patients with dementia was high in Korea. Approximately half of the patients with dementia received at least one DPIM with multiple DPIMs frequently used in these patients. Benzodiazepines were most commonly prescribed, followed by anticholinergics, H2RAs, and zolpidem. Overall, DPIMs were more likely to be prescribed in female patients using polypharmacy with multiple comorbidities, particularly Parkinson’s disease, mood disorder, and schizophrenia. Therefore, implementation of evidence-based public health policy as well as system-wide interventions such as close patient monitoring, medication reconciliation, and medication therapy management should be considered to minimize unnecessary DPIM use and ultimately, to prevent potential harms associated with DPIM use in geriatric patients with dementia.

## Figures and Tables

**Table 1:** List of potentially inappropriate medications in elderly patients with dementia (DPIMs) based on the institutional formulary according to the 2015 Beers criteria.

Drugs with Strong Anticholinergic Properties	
**Antidepressants**	**Antihistamines**
Amitriptyline Amoxapine Clomipramine Imipramine Nortriptyline Paroxetine	Chlorpheniramine Cyproheptadine Dimenhydrinate Hydroxyzine Triprolidine
**Antimuscarinics**	**Antipsychotics**
Fesoterodine Oxybutynine Solifenacin Trospium	Chlorpromazine Clozapine Olanzapine Perphenazine
**Antiparkinsonian**	**Antispasmodics**
Benztropine Trihexyphenidyl	Scopolamine
**Benzodiazepines**	
Alprazolam Bromazepam Chlordiazepoxide Clonazepam Clorazepate Clotiazepam	Diazepam Etizolam Flurazepam Loflazepate Lorazepam Triazolam
**H_2_-receptor antagonists**	
Cimetidine Famotidine Lafutidine	Ranitidine Roxatidine
**Nonbenzodiazepine, benzodiazepine receptor agonist hypnotics**	
Zolpidem	

**Table 2:** Baseline characteristics of patients included in this study ^a^.

Characteristics	DPIM Users (*n* = 987)	DPIM Non-Users (*n* = 1113)	Overall Patients (*n* = 2100)	*p*-Value ^b^
Age (years)	76 (73–80)	78 (73–82)	77 (73–81)	<0.001 ^c^
Sex				0025 ^d^
Male [*n* (%)]	339 (33.4)	435 (39.1)	774 (36.9)	
Female [*n* (%)]	648 (65.7)	678 (60.9)	1326 (63.1)	
Number of concurrent medications	8 (6–11)	4 (2–6)	6 (3–9)	<0.001 ^c^
Number of comorbid diseases	6 (4–8)	4 (2–6)	5 (3–7)	<0.001 ^c^
Concomitant diseases				
Parkinson’s disease [*n* (%)]	361 (36.6)	157 (14.1)	518 (24.7)	<0.001 ^d^
Mood (affective) disorder ^e^ [*n* (%)]	421 (42.7)	319 (28.7)	740 (35.2)	<0.001 ^d^
Schizophrenic disorder ^f^ [*n* (%)]	37 (3.7)	12 (1.1)	49 (2.3)	<0.001 ^d^
Gastrointestinal disorder ^g^ [*n* (%)]	228 (23.1)	179 (16.1)	407 (19.4)	<0.001 ^d^

^a^ Data presented as median (IQR) unless otherwise stated; ^b^ Comparison between DPIM users and non-users; ^c^ *p* value from Mann-Whitney *U* test; ^d^ *p* value from χ^2^ test; ^e^ Includes bipolar and depressive disorders; ^f^ Includes schizophrenia, schizotypal, and delusional disorders; ^g^ Includes diseases of esophagus, stomach, and duodenum.

**Table 3:** Prevalence of DPIM use in the study population (*n* = 2100).

Characteristics	Number (%) Unless Otherwise Stated
Patients with ≥ 1 DPIM prescription	987 (47.0)
Patients with ≥ 2 DPIM prescriptions	432 (20.6)
2 DPIMs	288 (13.7)
3 DPIMs	96 (4.6)
4 DPIM	31 (1.5)
≥5 DPIMs	17 (0.8)
Relative quantity of DPIM use (%, median [IQR])	14.3 [8.1–24.2]
Type of DPIMs	
Anticholinergics ^a^	555 (26.4)
Benzodiazepines	601 (28.6)
H2-receptor antagonists	146 (7.0)
Zolpidem	92 (4.4)

^a^ Drugs with strong anticholinergic properties as listed in Table 1.

**Table 4:** Univariate and multivariate analyses for the association between DPIM use and patient characteristics.

Patient Characteristics	Univariate Analysis		Multivariate Analysis	
	OR (95% CI)	*p*-Value	OR (95% CI)	*p*-Value
Female sex	1.226 (1.026–1.466)	0.025	1.355 (1.103–1.664)	0.004
Age	0.969 (0.956–0.983)	<0.001	0.989 (0.973–1.005)	0.181
Polypharmacy use ^a^	8.881 (7.110–11.093)	<0.001	5.146 (3.912–6.768)	<0.001
Number of comorbid diseases	1.385 (1.332–1.440)	<0.001	1.129 (1.073–1.188)	<0.001
Presence of:				
Parkinson’s disease	3.511 (2.839–4.344)	<0.001	1.799 (1.416–2.286)	<0.001
Mood (affective) disorder ^b^	1.851 (1.545–2.219)	<0.001	1.373 (1.111–1.695)	<0.001
Schizophrenia	3.573 (1.853–6.892)	<0.001	4.116 (1.959–8.648)	<0.001
Gastrointestinal disorder	1.567 (1.261–1.949)	<0.001	0.845 (0.651–1.099)	0.209

^a^ Number of concurrent medications ≥ 5; ^b^ Include bipolar and depressive disorders.

## Data Availability

The datasets generated and/or analyzed during the current study are not publicly available due to the inclusion of private medical information but may be available from the corresponding author upon reasonable request.
